# Tousled-like kinase 1: a novel factor with multifaceted role in mCRPC progression and development of therapy resistance

**DOI:** 10.20517/cdr.2021.109

**Published:** 2022-01-19

**Authors:** Md Imtiaz Khalil, Arrigo De Benedetti

**Affiliations:** Department of Biochemistry and Molecular Biology, LSU Health Sciences Center, Shreveport, LA 71103, USA.

**Keywords:** TLK1, NEK1, YAP1, VDAC1, DNA damage response, prostate cancer, metastatic castration-resistant prostate cancer, drug resistance in mCRPC

## Abstract

Standard treatment for advanced Prostate Cancer (PCa) consists of androgen deprivation therapy (ADT), but ultimately fails, resulting in the incurable phase of the disease: metastatic castration-resistant prostate cancer (mCRPC). Targeting PCa cells before their progression to mCRPC would greatly improve the outcome, if strategies could be devised selectively targeting androgen receptor (AR)-dependent and/or independent compensatory pathways which promote mCRPC development. Combination therapy by targeting the DNA damage response (DDR) along with ADT has been limited by general toxicity, and a goal of clinical trials is how to target the DDR more specifically. In recent years, our lab has identified a key role for the DDR kinase, TLK1, in mediating key aspects of adaptation to ADT, first by promoting a cell cycle arrest (through the TLK1>NEK1>ATR>Chk1 kinase cascade) under the unfavorable growth conditions (androgen deprivation), and then by reprogramming the PCa cells to adapt to androgen-independent growth via the NEK1>YAP/AR>CRPC conversion. In addition, TLK1 plays a key anti-apoptotic role via the NEK1>VDAC1 regulation on the intrinsic mitochondrial apoptotic pathway when the DDR is activated. Finally, TLK1 was recently identified as having an important role in motility and metastasis via regulation of the kinases MK5/PRAK and AKT (indirectly via AKTIP).

## INTRODUCTION

Prostate cancer (PCa) is one of the most common malignancies among men in the United States. Although death from PCa has been significantly reduced during the past two decades due to the advent of first (flutamide, nilutamide, cyproterone acetate, and bicalutamide) and second-generation (abiraterone acetate, enzalutamide, apalutamide, and recently approved darolutamide) anti-androgens, recent data of modeled projections suggests that the death rate is gradually increasing^[1-4]^. This might partly be due to the development of increased resistance towards androgen deprivation therapy (ADT), which eventually progresses to castration-resistant phenotype during which therapeutic options become limited. PCa is mainly a disease of uncontrolled proliferation of luminal epithelial cells of the prostate gland, although in some cases, basal cells are involved. Since androgen receptor (AR) signaling regulates the growth, survival, and proliferation of prostate tumors, the majority of PCa therapies are focused on either inhibition of androgen synthesis or blockade of androgen receptor transactivation. However, the drug effect does not last long, and the tumor relapses within 18-24 months with more aggressive phenotype known as castration-resistant prostate cancer (CRPC), even in the presence of castrate level of circulatory androgen. Besides androgen ablation, radiotherapy and chemotherapy are commonly employed for the treatment of both localized PCa and CRPC. Inflicting DNA damage and enhancing apoptosis of cancer cells are the mechanistic strategy of all radiotherapeutic (e.g., external beam radiation therapy, brachytherapy, radium-223) and some chemotherapeutic (PARP inhibitors, e.g., olaparib, niraparib, talazoparib; topoisomerase inhibitors, e.g., mitoxantrone, campothecin; platinum-based therapy, e.g., satraplatin, carboplatin, cisplatin; DNA crosslinking agent, e.g., mitomycin C) interventions (reviewed in Ref.^[5,6]^). It is observed that AR signaling is still active in the majority of CRPC due to the amplification, mutation, overexpression, and alternative splicing of AR that directly regulates the expression of DNA repair genes^[5,7-10]^. Therefore, a combinatorial treatment including ADT and other DNA damage-inducing agents should result in favorable clinical outcome and increase patients’ survival. Despite the improvement of treatment modalities, CRPC remains incurable, and drug-refractory PCa is evident among patients. Interestingly, other compensatory oncogenic survival mechanisms might play a role in conjunction or independent of AR signaling. Several receptor tyrosine kinases and mitogen-activated protein kinases mediated signaling are found to be activated in CRPC, which can bypass AR requirement for PCa cell growth and proliferation^[11-13]^. In this communication, we will focus on a less commonly reported serine/threonine kinase, TLK1 and its compensatory role in developing therapy resistance and metastatic CRPC (mCRPC) progression.

## THE MAMMALIAN TOUSLED LIKE KINASES AND TRANSLATIONAL UPREGULATION OF TLK1B

Mammalian homologues of plant tousled-kinase, known as tousled-like kinases (TLKs), were first cloned in 1999 and found to be implicated in DNA replication as their activities peak during the S-phase of the cell cycle^[14]^. Two members of the tousled-like kinase family have been identified (TLK1 and TLK2), which were found to share similar substrates and have partly redundant functions in maintaining genomic integrity. Later on, detailed studies of these two proteins identified their individual functions in various cellular processes along with their shared ones. TLK1 is reported to directly play a role in DNA replication, transcription, cell cycle checkpoint control, DNA damage response and repair (reviewed in Ref.^[15-17]^). Although multiple isoforms of TLK1 are discovered in different tissues, a translationally controlled isoform TLK1B has been thoroughly characterized by our group. TLK1B is an N-terminal truncated spliced variant of TLK1, which possesses a long, GC rich 5” UTR and two short upstream open reading fames, characteristics of a weakly competitive mRNA. TLK1B possesses an intact C-terminal catalytic domain, and its substrates specificity is similar to TLK1. A schematic sequence alignment of TLK1 and TLK1B can be found in Ref.^[18]^; while the alignment of TLK1 and its homolog TLK2 can be found in Ref.^[16,19]^. Translation of TLK1B is facilitated by the overexpression of eIF4E or by the stress-related activation of the AKT-mTOR pathway, which leads to 4EBP1 hyperphosphorylation and release of eIF4E from its inhibitor^[18,20,21]^. Histone H3 was one of the first identified substrates which can be phosphorylated on serine 10 residue by TLK1B. Although some other kinases (Aurora B/Ipl1 kinase) may also phosphorylate H3-S10, TLK1B phosphorylation of Histone H3-S10 promotes proper chromosomal condensation during metaphase and confers radio-resistance as observed in normal mouse breast epithelial cells (MM3MG) and yeast^[22]^. Another direct substrate of TLK1/1B is Histone H3-H4 chaperone Asf1a and Asf1b, which can be phosphorylated on several residues at C-terminus and increase their affinity to bind to H3-H4 that enables nucleosomal assembly with newly synthesized DNA strand during replication and damage repair^[23,24]^. TLK1B is also reported to phosphorylate Rad9 of Rad9-Rad1-Hus1 (9-1-1) heterotrimeric complex that acts as a scaffold to hold the DNA at the damaged region and recruits DNA repair factors^[25]^. Following DNA repair, TLK1/1B mediated phosphorylation of Rad9 at S328 promotes its dissociation and disassembly from the 9-1-1 trimeric complex and resumes the cell cycle from DNA damaged induced checkpoint arrest^[26]^. Besides these well-validated substrates of TLK1/1B, a proteomic screening from our lab identified 165 binding partners of TLK1/1B from a pool of 9000 full-length human proteins^[27]^. The physiological functions of the majority of these interactions are still unknown.

TLKs play a central role in DNA damage response and repair and hence, are critical for cell survival. However, studies aimed at identifying TLKs’ role in prostate cancer progression and drug resistance are very limited. Notably, the *TLK1* gene was identified by co-expression analysis using WGCNA as a key driver of PCa, highly enriched among candidate genes collected from expression Quantitative Trait Loci, somatic copy number alterations and prognostic analyses^[28]^. We also recently observed from data mining that TLK1 expression at mRNA level is increased in metastatic and high grade prostatic adenocarcinoma compared to the low risk tumors^[29]^. Moreover, androgen deprivation translationally increases TLK1B level in LNCaP cells and a PDX mice model through an mTORC1 dependent mechanism without increasing TLK1 mRNA expression; and mTOR inhibition by rapamycin reverses this phenomenon^[30,31]^. This compensatory activation of mTOR during AR blockade might be due to the oncogenic activation of AKT. AR inhibition prevents PHLPP mediated dephosphorylation of AKT by downregulating the expression of FKBP5^[32,33]^. The immunophilin *FKBP5* (FK506-binding protein 5) is an androgen-responsive gene whose scaffolding activity is required for PHLPP to dephosphorylate AKT at S473, a site on which phosphorylation is necessary for full activation of AKT^[34,35]^. In addition, FKBP5 enables the pharmacologic activity of rapamycin by binding to it that allows the association of FKBP5-rapamycin complex to the FKBP5-rapamycin binding domain (FRB domain) of mTORC1. FKBP5-rapamycin binding to FRB directly inhibits mTORC1 by masking its substrates docking sites or dissociating the regulatory subunits from the mTORC1 complex (reviewed in Ref.^[36]^).

## ROLE OF TLK1 IN PCA PROGRESSION AND DRUG RESISTANCE

The functional implications of TLK1 overexpression and/or TLK1B translational upregulation have multi-faceted role in driving drug resistance and hence, promoting PCa aggressiveness. Our group first demonstrated that TLK1/1B can mediate DNA damage response (DDR) through NIMA-related kinase 1 (NEK1). TLK1/1B tightly binds and phosphorylates NEK1 both *in vitro* and *in vivo* and this association is strengthened during DNA damage events^[27]^. Several studies reported NEK1 as a crucial factor in mediating the DDR along with its functions in other cellular processes, like the regulation of mitosis. In response to genotoxic threats, NEK1 is translocated to DNA damage foci in the nucleus and triggers the cell cycle checkpoint by activating Chk1 and/or Chk2^[37,38]^. The connection between NEK1 and DDR became more convincing by the findings that NEK1 kinase activity is required for ATR-ATRIP stability and ATR activation, which, in turn, activates Chk1^[39]^. Although NEK1 activity increases upon DNA damages, the upstream regulator of NEK1 was not reported before Singh *et al*.^[27]^ published work. Singh *et al*.^[27]^ reported that TLK1/1B phosphorylates NEK1 at a novel T141 residue that is adjacent to the activation loop and converts it to a hyperactive form. Since both TLK1/1B and NEK1 catalytic functions peak during the same phases of the cell cycle (S and G_2_/M) and by DNA damage, it is likely that their activities are linked, i.e., TLK1/1B regulates the kinase activity of NEK1. Indeed, genetic depletion of NEK1 or overexpression of a NEK1 T141A variant or pharmacologic inhibition of TLK1/1B, all resulted in reduced activation of ATR and Chk1 and increased S phase duration, but decreased G_2_/M phase cells suggesting the elimination of the cell cycle checkpoint after H_2_O_2_ treatment^[27]^.

## TLK1B MEDIATED COMPLEMENTATION OF THE AR/DDR ACTIVATION LOOP

Inhibition of AR signaling by ADT leads to the downregulation of multiple key DNA repair enzymes involved in homologous recombination repair (HRR), non-homologous end joining (NHEJ), and mismatch repair (MMR) pathways, resulting in inefficient DNA repair, which leads to genomic instability^[9]^. Several independent studies identified DNA-PKc, Ku70 (key players of NHEJ), BRCA1, RAD54L, RMI2 (associated with HRR), MSH2, MSH6 (key players of MMR) as direct targets of AR^[5,7-9,40]^. When cells try to undergo mitotic division with excessively damaged DNA, they become vulnerable to apoptotic death. These liabilities may render prostate cancer cells particularly sensitive to inhibition of specific DDR pathways, such as PARP in homologous recombination DNA repair^[41]^ and Chk1 in cell cycle checkpoint and DNA repair^[42]^, creating opportunities for synthetic lethality^[43]^. However, PCa cells may develop a compensatory mechanism by activating mTOR and translational upregulation of TLK1B to promote castration-resistant growth. Regulation via the translational increase in TLK1B isoform abrogates the need for its *de novo* gene expression, which is a faster and more energy-efficient mechanism of genome protection by activating TLK1>NEK1>ATR>Chk1 axis^[30]^. Singh *et al*.^[30,31]^. demonstrated that combination treatment of anti-androgen (bicalutamide) and TLK1 inhibitor [Thioridazine (THD)] synergistically decreases the formation of androgen-independent (AI) colonies of LNCaP cells and LNCaP xenograft tumors and increase apoptotic rate than bicalutamide treatment alone. We have yet to test in xenografts whether combination with second-generation anti-androgens, like enzalutamide, will produce the same effects, although, from the theoretical standpoint, there is no reason to suspect it would be different. Biochemically, ADT+THD treatment reduces pNEK1 T141, pATR, and pChk1 level, which suggest the possible involvement of TLK1>NEK1>ATR>Chk1 mediated DDR pathway in the development of CRPC. Similarly, NEK1 T141A overexpressing LNCaP and xenograft tumor remain sensitive to ADT and do not transition to AI growth, which confirms the persistence of TLK1>NEK1 signaling in PCa drug resistance to ADT^[30]^. Correlated increase of TLK1B and pNEK1 T141 with PCa progression was evident from a human PCa-TMA, TRAMP (a PCa mice model) and PDX tumor tissues; all suggested the universality of TLK1>NEK1 signaling in PCa models^[31]^. In fact, targeting TLK1 could potentiate the activity of other DNA damaging agents as evident from our group and others’ works in different cancers. For instance, TLK1 inhibition chemosensitizes PCa (PC3) and breast cancer (MDA-MB-231 and 4T1) cells to doxorubicin (Ronald *et al*.^[44]^; Jin *et al*.^[45]^), glioblastoma cells (U87MG) to temozolomide (Ibrahim *et al*.^[46]^), cholangiocarcinoma (SSP25 and HuCCT1) and ovarian cancer cells (SKOV-3) to cisplatin (Takayama *et al*.^[47]^; Rho *et al*.^[48]^). Since resistance to cisplatin, doxorubicin, and radiotherapies are common in CRPC, TLK1 inhibition may reverse the resistance of CRPC cells to these agents^[49,50]^. Our group was first to identify a number of compounds that inhibit TLK1 and TLK2 in the class of phenothiazines antipsychotics, suggesting their repurposing as a clinical option^[44]^, and a newer derivative (J54) lacking appreciable anti-dopaminergic activity^[51]^.

## ROLE OF TLK1 IN THE HIPPO/YAP PATHWAY

TLK1-NEK1 signaling has pleotropic effects on several oncogenic processes regulating PCa progression and drug resistance. A key finding from our lab is that TLK1 dependent hyperactive NEK1 can phosphorylate YAP and promote its stability^[52]^. The observation that NEK1 can interact and phosphorylate TAZ prompted us to investigate whether NEK1 can also phosphorylate YAP, a key effector of hippo pathway highly homologous to TAZ^[53]^. NEK1 genetic depletion or NEK1 T141A variant overexpression or TLK1 inhibition all resulted in YAP degradation and downregulation of YAP target genes in PCa cells. In addition, we demonstrated that NEK1 can phosphorylate YAP1 in six unique residues *in vitro*, of which pYAP1 Y407 seems most interesting^[52]^. YAP1 Y407 phosphorylation by NEK1 may increase the binding affinity of YAP to its transcriptional activators resulting in YAP nuclear translocation away from cytoplasmic degradation. Determining the functional relevance of these phosphorylation sites of YAP in CRPC progression and drug resistance development is currently an active pursuit of our lab. TLK1>NEK1>YAP axis may also converge with the reciprocal feedback loop of AR and DDR (AR signaling induces DNA repair genes expression and DNA damage increases AR activity), as YAP regulates AR target genes expression^[7,54,55]^.

## ROLE OF TLK1 IN OTHER PATHWAYS

TLK1-NEK1 axis regulates other survival pathways independent of DDR and hippo/YAP regulation. We demonstrated that TLK1>NEK1 axis can promote survival of PCa cells by maintaining mitochondrial membrane integrity by phosphorylating VDAC1 leading to apoptotic suppression^[56]^. VDAC1 is a pore-forming, outer mitochondrial membrane protein that regulates metabolic and ionic exchanges between the mitochondria and cell and is often overexpressed in high grade PCa (reviewed in Ref.^[57]^). NEK1 can phosphorylate VDAC1 on S193 residue controlling its gatekeeping activity^[58]^. We demonstrated that disruption of TLK1>NEK1 axis sensitizes the PCa cells to low doses of doxorubicin treatment by reducing VDAC1 S193 phosphorylation and its stability. Doxorubicin treatment of NEK1 T141A mutant variant overexpressing cells displayed enhanced population of sub G1 cells, reduced oxygen consumption, and increased cytochrome C (Cyt-C) release in cytoplasm resulting in the activation of intrinsic apoptotic pathway compared to the wild type NEK1 overexpressing cells^[56]^.

**Figure 1:**
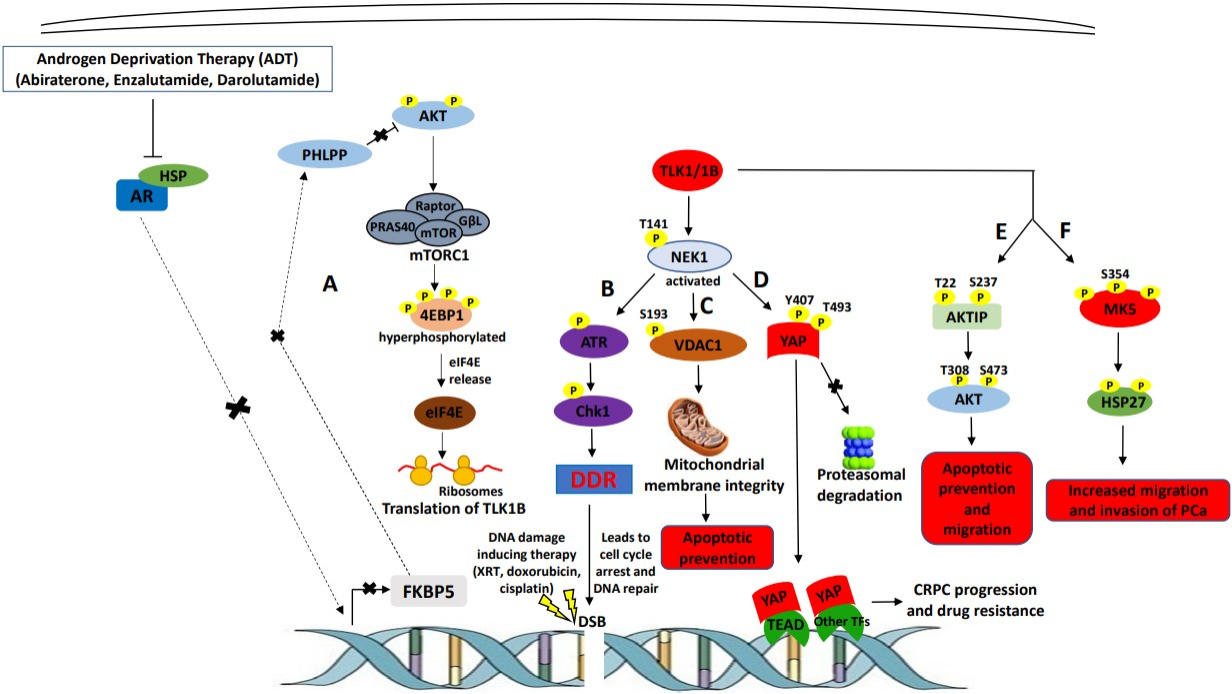
TLK1’s role in mediating mCRPC progression and therapy resistance. (A) androgen deprivation therapy (ADT) blocks androgen receptor (AR) nuclear localization resulting in FKBP5 downregulation, which in turn, leads to AKT activation due to reduced dephosphorylation activity of PHLPP. AKT activation leads to mTORC1 activation, which activates 4EBP1 and releases eIF4E. Excess eIF4E initiates the translation of TLK1B. (B) TLK1/1B activates NEK1 by T141 phosphorylation, which in turn, activates ATR>Chk1>DNA damage response (DDR) signaling cascade. Activation of DDR promotes DNA repair inflicted by DNA damaging therapeutic agents. (C) TLK1>NEK1 axis is involved in VDAC1 S193 phosphorylation which maintains mitochondrial membrane integrity and suppresses intrinsic apoptotic signaling. (D) TLK1>NEK1 signaling phosphorylates YAP on Y407 and T493 residues and promotes the stabilization of YAP by binding to TEAD or other transcription factors (TF), leading to nuclear relocalization escaping proteasomal degradation. YAP stabilization and accumulation lead to CRPC progression and drug resistance. (E) TLK1 directly phosphorylates AKTIP on T22 and S237 residues, which stimulates AKT phosphorylation and activation, resulting in pro-survival and pro-migratory signaling. (F) TLK1 directly interacts and phosphorylates MK5, which increases its catalytic activity towards HSP27 (an MK5 substrate) and leads to increased migration and invasion, hence, metastasis of PCa cells.

TLK1 also directly facilitates other pro-survival pathways through some substrates independent of NEK1. TLK1 mediates anti-apoptotic responses through AKT activation by interacting and phosphorylating AKT interacting protein (AKTIP). TLK1 phosphorylation of AKTIP on T22 and S237 residues increases its scaffolding activity to anchor both AKT and PDK1 to the phosphatidylinositol (3,4,5)-trisphosphate (PIP3) regions of the plasma membrane where PDK1 can phosphorylate AKT on T308 residue followed by S473 phosphorylation by mTORC2 complex for full activation of AKT^[59]^. Fully activated AKT may relay pro-survival signals by inhibiting BAD, caspase 9, and FoxO3 pro-apoptotic factors through phosphorylation facilitating CRPC progression. However, the role of AKTIP phosphorylation by TLK1 in CRPC drug resistance needs to be further assessed. In a recent endeavor, we uncovered the novel interaction between TLK1 and MK5 that promotes PCa cell motility and invasion. MK5 is a known promotility kinase that can be phosphorylated by TLK1 in three unique residues that increases MK5’s catalytic function. We demonstrated that one phospho-acceptor site of MK5 (S354) by TLK1 is present in all major PCa cell lines of both androgen-dependent and independent categories. Furthermore, ADT increases pMK5 S354 level in LNCaP cells, and this pMK5 protein is progressively increased in tumors of higher grades and nodal metastatic scores analyzed from TRAMP mice prostate tissue and human PCa TMA, suggesting that the TLK1>MK5 signaling may be associated with increased aggressiveness of PCa. Finally, disruption of TLK1>MK5 axis by TLK1 or MK5 inhibition drastically reduces PCa cells motility analyzed in a panel of PCa cell lines^[29]^. Figure 1 graphically depicts the currently known multifaceted roles of TLK1 in mCRPC progression and acquisition of drug resistance.

## TARGETING TLK1 AND THE DDR TO REDUCE DRUG RESISTANCE

DNA damage response (DDR) is a vital mechanism for PCa cells’ survival and propagation. In addition, intact DDR activity may give hormone-refractory PCa cells an undue advantage against DNA damage-inducing therapies by keeping their genomic instability to sub-toxic level, thus, promoting drug resistance. Targeting TLK1 might be an excellent approach to impair DDR along with other oncogenic survival pathways as mentioned earlier. TLK1 is a better druggable target than other kinases of the DDR pathway, such as ATM or Chk1, to reduce general toxicity as ADT selectively increases TLK1B level in PCa cells, which can be easily manipulated using TLK1 specific inhibitors sparing the non-cancerous cells. Further efforts need to be given to design and generate better TLK1 inhibitors with improved pharmacokinetics, safety, and tolerability for clinical application. In fact, in recent years, our lab, in collaboration with others, has designed and synthesized a phenothiazine analog named J54 with higher efficacy in TLK1 inhibition and lower non-target effects unlike THD^[51,52]^. Personalized application of TLK1 inhibition as an adjuvant and neoadjuvant therapy in combination with other PCa directed treatments may overcome the drug-resistant phenotype of CRPC and bring therapeutic benefit.

## DECLARATIONS

### Availability of data and materials

Description of data and materials can be found in the referenced article. No new data are included in this review.

### Copyright

© The Author(s) 2022.
